# Supplementary material for: Conservation of regulatory elements with highly diverged sequences across large evolutionary distances
Source: Nat Genet. 2025 May 27;57(6):1524–34. doi: 10.1038/s41588-025-02202-5 (PMC12165850; doi:10.1038/s41588-025-02202-5)
Supplement: Supplementary file 1 — Supplementary Note. [file 41588_2025_2202_MOESM1_ESM.pdf]

nature genetics

Supplementary information https://doi.org/10.1038/s41588-025-02202-5

Conservation of regulatory elements with highly diverged sequences across large evolutionary distances

In the format provided by the authors and unedited

Table of Content

Interspecies Point Projection Algorithm its Usage and Parameterization P.2

Interspecies Point Projection (IPP)

We project genomic point coordinates from a reference genome to a target genome by linear interpolation between blocks of pairwise sequence alignment, so called anchor points [43]. Moreover, we use pairwise alignments between a set of bridging species to maximize anchor point density and thus optimize projection accuracy. This scenario is represented by a graph in which every node is a species, and the weighted edges represent the distance of a genomic coordinate to its anchor points between the nodes it connects (Fig. 2). We established a distance scoring function that returns a score of 1 for a genomic location x overlapping an anchor point ($|x - a| = 0$), and exponentially converges to zero with increasing distance $|x - a|$. For a single pairwise comparison, the function is defined as follows:

$$f(x) = exp(-\frac{d_{min}}{g_R\, s}), \quad (1)$$

with $d_{min} = min\{|x - a^{(1)}|, |x - a^{(2)}|\}$ denoting the distance of a genomic location x to its closest anchor point, g_R denoting the genome size of the reference species and s a scaling factor that can be tweaked to determine the decreasing rate of the function. For instance, we can set s by defining a distance half life d_h as the distance $|x - a|$ at which the scoring function ought to return a value of 0.5:

$$s = -\frac{d_h}{g_B\ log(0.5)}. \quad (2)$$

All projections presented in this manuscript were computed using a distance half life of 10 kb.
For the score calculation in Equation 1, the distance is normalized by the genome size of the reference species (g_R) of a pairwise comparison. In Equation 2, the scaling factor is normalized by the size of a basis genome (g_B) which we chose to be the mouse genome build mm39, allowing comparisons between projections from different reference species. In practice, this means that the distance scoring function decreases at equal rates for different reference genomes, however, these scores correspond to different distances based on the relative reference genome sizes. The function can thus be simplified to the following form:

$$f(x) = 0.5^{(\frac{d_{min}}{d_h}\frac{g_B}{g_R})}. \quad (3)$$

We can then compute the total distance score of a given path through the graph as the product of the score of all edges in that path. The length of a path is reciprocal to the distance scoring function, hence we can subtract the total score from 1 to obtain the path length l_p:

$$l_p = 1 - \prod_{i\in p} f(x_i). \quad (4)$$

Finally, projection accuracy is optimized by finding the shortest path through the graph:

$$\dot{p} = arg\ \min_{p\in P} l_p, \quad (5)$$

with P denoting the set of all paths through the graph connecting the reference and the target species. Finding the shortest path through a graph can be solved using Dijkstra's Shortest Path Algorithm [63]. We implemented the method in python and C++ and named it Interspecies Point Projection (IPP). IPP is publicly available at https://github.com/tobiaszehnder/ipp .

Bridging species selection and pairwise alignment

IPP relies on the additional anchor points provided by bridging species to map corresponding genomic locations between pair of divergent genomes (**Extended Data Figure 2a,b**). As such, the choice of bridging species depends on the specific comparison of interest. Here, for a mouse-chicken comparison, we selected mammalian species which have diverged from chicken after mouse (human, pig, mole, opossum, platypus), and those which have diverged from mouse after chicken (alligator, green anole, snake, turtle) (**Supplementary Table**). Additionally, we selected the rat and emu as two closely related species to mouse and chicken, respectively. Finally, we included frogs, zebrafish, and the sea squirt as outgroups.
Fasta files for all reference genome assemblies were obtained either from DNA Zoo or from NBCI were used as inputs for pairwise alignments with *lastal*. Chain files were then generated, preprocessed, and merged for each species pair before combined in one collection of large pairwise alignments and stored in a binary format. This collection of alignments consisting of the reference, target, and all bridging species is the necessary input for running IPP.

Projection classification and distance score tuning

IPP computes a score for every projection from one genome to another through the species graph. As described above, this score is a representation of the distance to the nearest anchor points, i.e. the higher the score, the shorter the distance and thus the more accurate the projection. We use this distance score as a threshold to classify projections into 3 classes: directly-conserved (DC), indirectly-conserved (IC), and non-conserved (NC) (**Extended Data Fig. 2c**). An element is classified as *DC* if its projection score using only direct alignments was above this threshold, and as *IC* if their projection score from bridging alignment is also above such threshold. All remaining elements are then classified as *NC*. If ATAC-Seq data is available for the target genome, we further classify each projection by their functional conservation. Specifically, any projected point is classified as functionally conserved/'**+**' or non-functionally conserved/'**-**', if it is within or outside a 1,500bp distance from an ATAC-seq peak summit, respectively.

Initially, we set the score threshold at 0.99 which, given Equation 2, represents a maximum distance to the next anchor point of ~150 bps for DC elements. For IC elements, this means that a) the projection required an intermediate species, and b) the sum of distances from the query element to an anchor point at all intermediate projections is =< 150bp. While ensuring the confidence of projections, this very stringent cutoff implies a high level of false negatives within NC, i.e. projections with a projection score below the cutoff that are nevertheless pointing to the correct ortholog. Furthermore, taking into practical consideration that IPP only maps a single base-pair of an element between genomes, such stringency likely results in an underestimation of conservation of the element of interest.

We then sought to tune this threshold parameter, which is ultimately a trade-off between specificity and sensitivity. In other words, relaxing the distance threshold will result in more elements being classified as conserved with a higher likelihood of such classification being a false positive (i.e. a projection pointing to a non-orthologous region. We took advantage of available ATAC-Seq data in the chicken forelimb as an independent tissue model and determine if and how the proportion of functionally conserved elements changes as we relax the cut-off score. We observe a clear drop in the fraction of functionally conserved elements at high projection scores (i.e. >=0.9) from 38% to below 27% of all conserved enhancers (**Extended Data Fig. 2d**). This sharp change in proportion appears to plateau at lower projection score thresholds. Indeed, even with dramatically forgiving cutoffs, just over 20% of all projections is putatively functionally conserved at every score threshold below 0.75. Importantly, this trend is not reflective of the spatial distribution of open chromatin, as only ~10% of randomly selected background regions reside within open chromatin after projection (**Extended Data Fig.2d**).

Given the availability of equivalent functional datasets, we decided to relax PS cut-off and used 2 different distance cut-offs for DC and IC classifications. Specifically, an element with a PS of 0.979 (~300bp distance) using only direct alignments is classified as DC. For IC classification, we used a score cut-off of 0.841, which is equivalent to a summed distance of 2.5kb through all intermediate projections. These projections are filtered - as before - for those overlapping open chromatin regions to select for putatively functionally conserved elements.

An alternative approach to identify functionally conserved orthologs:

One can take advantage of a very relaxed approach to identify putative *functionally* conserved orthologous enhancers (e.g. projections residing in open chromatin), providing a complementary layer of functional validation such as ATAC-seq. By simply ommitting the lower (IC/NC) cut-off, all "+"-projections would be identified and IC-projections could be discarded. This permits the detection of functional orthologs in highly dynamic genomic neighborhoods where sequence alignments are sparse, with the potential cost of a higher false discovery rate.
