# Supplementary material for: Conservation of regulatory elements with highly diverged sequences across large evolutionary distances
Source: Nat Genet. 2025 May 27;57(6):1524–34. doi: 10.1038/s41588-025-02202-5 (PMC12165850; doi:10.1038/s41588-025-02202-5)
Supplement: Supplementary file 2 — Reporting Summary [file 41588_2025_2202_MOESM2_ESM.pdf]

nature portfolio

Corresponding author(s): Ibrahim, Daniel

Last updated by author(s): Mar 9, 2025

Reporting Summary

Nature Portfolio wishes to improve the reproducibility of the work that we publish. This form provides structure for consistency and transparency in reporting. For further information on Nature Portfolio policies, see our Editorial Policies and the Editorial Policy Checklist.

Statistics

For all statistical analyses, confirm that the following items are present in the figure legend, table legend, main text, or Methods section.

n/a	Confirmed	
☐	☒	The exact sample size (*n*) for each experimental group/condition, given as a discrete number and unit of measurement
☒	☐	A statement on whether measurements were taken from distinct samples or whether the same sample was measured repeatedly
☐	☒	The statistical test(s) used AND whether they are one- or two-sided<br>*Only common tests should be described solely by name; describe more complex techniques in the Methods section.*
☒	☐	A description of all covariates tested
☒	☐	A description of any assumptions or corrections, such as tests of normality and adjustment for multiple comparisons
☐	☒	A full description of the statistical parameters including central tendency (e.g. means) or other basic estimates (e.g. regression coefficient) AND variation (e.g. standard deviation) or associated estimates of uncertainty (e.g. confidence intervals)
☐	☒	For null hypothesis testing, the test statistic (e.g. *F*, *t*, *r*) with confidence intervals, effect sizes, degrees of freedom and *P* value noted<br>*Give P values as exact values whenever suitable.*
☒	☐	For Bayesian analysis, information on the choice of priors and Markov chain Monte Carlo settings
☒	☐	For hierarchical and complex designs, identification of the appropriate level for tests and full reporting of outcomes
☐	☒	Estimates of effect sizes (e.g. Cohen's *d*, Pearson's *r*), indicating how they were calculated

Our web collection on statistics for biologists contains articles on many of the points above.

Software and code

Policy information about availability of computer code

Data collection	No software was used to collect data
Data analysis	Details about analysis are provided in the Methods section.<br>Code to run IPP can be found at https://github.com/tobiaszehnder/IPP<br><br>Softwares used to process sequencing libraries:<br>- samtools v1.10<br>- bowtie2 v.2.3.5<br>- deepTools<br>- BWA-mem v0.7.17<br>- cutadapt v 1.10<br>- Juicer v1.6.0<br>- STARv2.7.9a<br>- Genrich v0.6.1 https://github.com/jsh58/Genrich<br>- Picard v2.23.4<br>- FANC 0.9.25<br>- CRUP https://github.com/VerenaHeinrich/CRUP<br>- TOBIAS https://github.com/loosolab/TOBIAS<br><br>Main R packages used (for R version 4.2.1)<br>- tidyverse 1.3.2

- rtracklayer 1.58
- DESeq2 1.36
- universalmotif 1.14.1
- clusterProfiler 4.4.4
- memes 1.4.1
- BSgenome 1.66.3
- rankdist 1.1.4
- biomaRt 2.52.0
- gkmSVM 0.83

Softwares for building SVM model (Python 3.8.5)
- lsgkm https://github.com/kundajelab/lsgkm-svr
- GkmExplain https://github.com/kundajelab/lsgkm-svr
- TFMoDisco https://github.com/jmschrei/tfmodisco-lite

For manuscripts utilizing custom algorithms or software that are central to the research but not yet described in published literature, software must be made available to editors and reviewers. We strongly encourage code deposition in a community repository (e.g. GitHub). See the Nature Portfolio guidelines for submitting code & software for further information.

Data

Policy information about availability of data

All manuscripts must include a data availability statement. This statement should provide the following information, where applicable:

- Accession codes, unique identifiers, or web links for publicly available datasets
- A description of any restrictions on data availability
- For clinical datasets or third party data, please ensure that the statement adheres to our policy

ChIPmentation, RNA-seq, ATAC-seq and Hi-C sequencing data generated from chicken embryonic heart and forelimb and mouse embryonic heart ATAC-seq data study have been deposited to NCBI GEO under GSE263587, GSE263753, GSE263755, GSE263783.
Following published dataset were re-analysed with details listed in Sup Table 2(GSM2544836, GSE185775, GSE185775, GSE185775, GSE185775, GSE185775, ENCSR582SPN, ENCSR266JQW, ENCSR782DGO, ENCSR782DEA, GSE185775, GSE185775, GSE185775, GSE185775, GSE185775, GSE185775, ENCSR222IHX, GSE185775, ENCSR963OLG, GSE185775, ENCSR886IHN, ENCSR592GQI, GSE164737, GSE164738)

Research involving human participants, their data, or biological material

Policy information about studies with human participants or human data. See also policy information about sex, gender (identity/presentation), and sexual orientation and race, ethnicity and racism.

Reporting on sex and gender	not applicable
Reporting on race, ethnicity, or other socially relevant groupings	not applicable
Population characteristics	not applicable
Recruitment	not applicable
Ethics oversight	not applicable

Note that full information on the approval of the study protocol must also be provided in the manuscript.

Field-specific reporting

Please select the one below that is the best fit for your research. If you are not sure, read the appropriate sections before making your selection.

☒ Life sciences ☐ Behavioural & social sciences ☐ Ecological, evolutionary & environmental sciences

For a reference copy of the document with all sections, see nature.com/documents/nr-reporting-summary-flat.pdf

Life sciences study design

All studies must disclose on these points even when the disclosure is negative.

Sample size	No prior analyses were used to determine the sample size of 2 biological replicates per ChIP/ATAC/RNA-seq and Hi-C experiment, but are based on standards in the field. Testing of 3 or more lacZ transgenic embryos from independent tetraploid aggregations was determined to generate sufficient embryos for lacZ stainings

Data exclusions	The embryos that were not at the correct developmental stage were excluded from data collection.
Replication	For lacZ enhancer-reporter assays, at least 3 animals/embryos of the appropriate genotype were stained and produced reproducible staining/ phenotypes. Functional genomic experiments were produced in two biological replicates for each species and developmental stage
Randomization	There was no randomization of samples in this study
Blinding	Blinding was not relevant for our study, as enhancer-Reporter assays needed meticulous tracing of plasmids, cell cultures, and foster mothers to avoid mix-up. Consequently formal blinding of the experimental result was not possible. The resutls of the biological samples are not impacted by the unblinded design

Reporting for specific materials, systems and methods

We require information from authors about some types of materials, experimental systems and methods used in many studies. Here, indicate whether each material, system or method listed is relevant to your study. If you are not sure if a list item applies to your research, read the appropriate section before selecting a response.

Materials & experimental systems

n/a	Involved in the study
☐	☒ Antibodies
☐	☒ Eukaryotic cell lines
☒	☐ Palaeontology and archaeology
☐	☒ Animals and other organisms
☒	☐ Clinical data
☒	☐ Dual use research of concern
☒	☐ Plants

Methods

n/a	Involved in the study
☐	☒ ChIP-seq
☒	☐ Flow cytometry
☒	☐ MRI-based neuroimaging

Antibodies

Antibodies used	H3K4me1 (Diagenode #C15410037), H3K4me3 (Merck-Millipore #07-473), H3K27ac (Diagenode #C15410174)
Validation	Antibodies were validated in independent ChIP experiments on the manufacturer's website using ChIP-qPCR, ChIP-seq, Western Blots, Immunfluorescence and ELISAs.

Eukaryotic cell lines

Policy information about cell lines and Sex and Gender in Research

Cell line source(s)	G4-ESCs were obtained from Anders Nagy and subsequently used to generate custom genome-engineered cell lines for generation of mice used in this study.
Authentication	The pluripotent state of the ESCs used was authenticated by generation of highly chimeric, germ-line transmitting mice through di- and tetraploid complementation assays
Mycoplasma contamination	all cell lines were tested negative for mycoplasma contamination
Commonly misidentified lines (See ICLAC register)	No commonly misidentified cell lines were used

Animals and other research organisms

Policy information about studies involving animals; ARRIVE guidelines recommended for reporting animal research, and Sex and Gender in Research

Laboratory animals	Mouse lines described in this study were C57Bl.6/J mice genotype and chicken material was obtained as fertilized SPF eggs purchased from Valo Biomedia.
Wild animals	not applicable
Reporting on sex	Sex was not determined for embryo collection, but cohorts were presumed to include roughly equal numbers of males and females.
Field-collected samples	not applicable
Ethics oversight	The study plan was approved by the Landesamt für Gesundheit und Soziales (LaGeSo), Berlin under licenses G0243/18 and G0098/23.

Note that full information on the approval of the study protocol must also be provided in the manuscript.

Plants

Seed stocks	not applicable
Novel plant genotypes	not applicable
Authentication	not applicable

ChIP-seq

Data deposition

- [x] Confirm that both raw and final processed data have been deposited in a public database such as GEO.
- [x] Confirm that you have deposited or provided access to graph files (e.g. BED files) for the called peaks.

Data access links
May remain private before publication.

ChIPmentation, RNA-seq, ATAC-seq and Hi-C sequencing data generated from chicken embryonic heart and forelimb and mouse embryonic heart ATAC-seq data study have been deposited to NCBI GEO under GSE263587, GSE263753, GSE263755, GSE263783.

Files in database submission

H3K4me3_heart_HH24_galGal6_WT_Rep2_R2_001.fastq.gz
H3K4me3_heart_HH24_galGal6_WT_Rep2_R1_001.fastq.gz
H3K27ac_heart_HH22_galGal6_WT_Rep2_R1_001.fastq.gz
H3K4me1_heart_HH22_galGal6_WT_Rep1_R1_001.fastq.gz
H3K27ac_heart_HH22_galGal6_WT_Rep2_R2_001.fastq.gz
H3K4me1_heart_HH22_galGal6_WT_Rep1_R2_001.fastq.gz
input_heart_HH24_galGal6_WT_Rep1_R1_001.fastq.gz
H3K4me3_FL_HH22_galGal6_WT_Rep1_R1_001.fastq.gz
H3K4me3_FL_HH22_galGal6_WT_Rep1_R2_001.fastq.gz
H3K4me3_FL_HH24_galGal6_WT_Rep1_R2_001.fastq.gz
H3K27ac_heart_HH22_galGal6_WT_Rep1_R2_001.fastq.gz
H3K4me3_heart_HH22_galGal6_WT_Rep1_R2_001.fastq.gz
H3K4me3_heart_HH22_galGal6_WT_Rep1_R1_001.fastq.gz
H3K4me3_FL_HH24_galGal6_WT_Rep1_R1_001.fastq.gz
H3K4me1_heart_HH24_galGal6_WT_Rep2_R2_001.fastq.gz
H3K27ac_heart_HH24_galGal6_WT_Rep2_R2_001.fastq.gz
H3K27ac_heart_HH22_galGal6_WT_Rep1_R1_001.fastq.gz
H3K27ac_heart_HH24_galGal6_WT_Rep2_R1_001.fastq.gz
H3K27ac_FL_HH24_galGal6_WT_Rep1_R2_001.fastq.gz
H3K27ac_heart_HH24_galGal6_WT_Rep1_R2_001.fastq.gz
H3K27ac_heart_HH24_galGal6_WT_Rep1_R1_001.fastq.gz
H3K4me1_heart_HH24_galGal6_WT_Rep2_R1_001.fastq.gz
H3K27ac_FL_HH24_galGal6_WT_Rep1_R1_001.fastq.gz
H3K4me1_heart_HH22_galGal6_WT_Rep2_R1_001.fastq.gz
H3K4me1_heart_HH22_galGal6_WT_Rep2_R2_001.fastq.gz
H3K4me1_FL_HH22_galGal6_WT_Rep1_R2_001.fastq.gz
H3K27ac_FL_HH22_galGal6_WT_Rep1_R2_001.fastq.gz
H3K27ac_FL_HH22_galGal6_WT_Rep1_R1_001.fastq.gz
H3K4me1_FL_HH22_galGal6_WT_Rep1_R1_001.fastq.gz
H3K4me3_heart_HH22_galGal6_WT_Rep2_R1_001.fastq.gz
H3K4me3_heart_HH22_galGal6_WT_Rep2_R2_001.fastq.gz
H3K4me1_FL_HH24_galGal6_WT_Rep1_R2_001.fastq.gz
H3K4me1_FL_HH24_galGal6_WT_Rep1_R1_001.fastq.gz
H3K4me1_heart_HH24_galGal6_WT_Rep1_R1_001.fastq.gz
H3K4me1_heart_HH24_galGal6_WT_Rep1_R2_001.fastq.gz
H3K4me3_heart_HH24_galGal6_WT_Rep1_R1_001.fastq.gz
H3K4me3_heart_HH24_galGal6_WT_Rep1_R2_001.fastq.gz
H3K4me1_FL_HH24_galGal6_WT_Rep2_R2_001.fastq.gz
H3K27ac_FL_HH24_galGal6_WT_Rep2_R1_001.fastq.gz
H3K4me1_FL_HH24_galGal6_WT_Rep2_R1_001.fastq.gz
H3K27ac_FL_HH24_galGal6_WT_Rep2_R2_001.fastq.gz
H3K4me3_FL_HH24_galGal6_WT_Rep2_R2_001.fastq.gz
H3K4me3_FL_HH24_galGal6_WT_Rep2_R1_001.fastq.gz
H3K4me3_FL_HH24_galGal6_WT_Rep2.cpm.bw
H3K27ac_FL_HH24_galGal6_WT_Rep2.cpm.bw

H3K27ac_heart_HH24_galGal6_WT_Rep1.cpm.bw
H3K4me3_heart_HH24_galGal6_WT_Rep2.cpm.bw
H3K4me1_FL_HH24_galGal6_WT_Rep2.cpm.bw
H3K4me3_heart_HH22_galGal6_WT_Rep2.cpm.bw
H3K4me1_heart_HH24_galGal6_WT_Rep2.cpm.bw
H3K4me1_heart_HH22_galGal6_WT_Rep2.cpm.bw
H3K4me3_FL_HH24_galGal6_WT_Rep1.cpm.bw
H3K4me3_heart_HH24_galGal6_WT_Rep1.cpm.bw
H3K4me1_FL_HH24_galGal6_WT_Rep1.cpm.bw
H3K4me3_FL_HH22_galGal6_WT_Rep1.cpm.bw
H3K4me3_heart_HH22_galGal6_WT_Rep1.cpm.bw
H3K27ac_FL_HH24_galGal6_WT_Rep1.cpm.bw
H3K27ac_heart_HH24_galGal6_WT_Rep2.cpm.bw
H3K27ac_heart_HH22_galGal6_WT_Rep2.cpm.bw
H3K4me1_heart_HH22_galGal6_WT_Rep1.cpm.bw
H3K4me1_heart_HH24_galGal6_WT_Rep1.cpm.bw
H3K27ac_heart_HH22_galGal6_WT_Rep1.cpm.bw
H3K27ac_FL_HH22_galGal6_WT_Rep1.cpm.bw
H3K4me1_FL_HH22_galGal6_WT_Rep1.cpm.bw
ATAC-seq_FL_HH22_galGal6_WT_Rep2_R1_001.fastq.gz
ATAC-seq_FL_HH22_galGal6_WT_Rep2_R2_001.fastq.gz
ATAC-seq_heart_HH24_galGal6_WT_Rep1_R2_001.fastq.gz
ATAC-seq_heart_HH24_galGal6_WT_Rep2_R2_001.fastq.gz
ATAC-seq_heart_HH24_galGal6_WT_Rep1_R1_001.fastq.gz
ATAC-seq_heart_HH24_galGal6_WT_Rep2_R1_001.fastq.gz
ATAC-seq_FL_HH24_galGal6_WT_Rep1_R2_001.fastq.gz
ATAC-seq_FL_HH24_galGal6_WT_Rep1_R1_001.fastq.gz
ATAC-seq_FL_HH24_galGal6_WT_Rep2_R2_001.fastq.gz
ATAC-seq_FL_HH24_galGal6_WT_Rep2_R1_001.fastq.gz
ATAC-seq_heart_E115_mm39_WT_Rep1_R2_001.fastq.gz
ATAC-seq_heart_E115_mm39_WT_Rep1_R1_001.fastq.gz
ATAC-seq_heart_E115_mm39_WT_Rep2_R2_001.fastq.gz
ATAC-seq_heart_E115_mm39_WT_Rep2_R1_001.fastq.gz
ATAC-seq_heart_E105_mm39_WT_Rep2_R1_001.fastq.gz
ATAC-seq_heart_E105_mm39_WT_Rep2_R2_001.fastq.gz
ATAC-seq_heart_E105_mm39_WT_Rep1_R2_001.fastq.gz
ATAC-seq_heart_E105_mm39_WT_Rep1_R1_001.fastq.gz
ATAC-seq_heart_HH22_galGal6_WT_Rep2_R2_001.fastq.gz
ATAC-seq_heart_HH22_galGal6_WT_Rep2_R1_001.fastq.gz
ATAC-seq_heart_HH22_galGal6_WT_Rep1_R1_001.fastq.gz
ATAC-seq_heart_HH22_galGal6_WT_Rep1_R2_001.fastq.gz
ATAC-seq_FL_HH22_galGal6_WT_Rep1_R2_001.fastq.gz
ATAC-seq_FL_HH22_galGal6_WT_Rep1_R1_001.fastq.gz
ATAC-seq_FL_HH22_galGal6_WT_Rep2.cpm.bw
ATAC-seq_heart_HH22_galGal6_WT_Rep2.cpm.bw
ATAC-seq_heart_HH22_galGal6_WT_Rep1.cpm.bw
ATAC-seq_FL_HH24_galGal6_WT_Rep2.cpm.bw
ATAC-seq_FL_HH24_galGal6_WT_Rep1.cpm.bw
ATAC-seq_heart_HH24_galGal6_WT_Rep1.cpm.bw
ATAC-seq_FL_HH22_galGal6_WT_Rep1.cpm.bw
ATAC-seq_heart_E105_mm39_WT_Rep2.cpm.bw
ATAC-seq_heart_HH24_galGal6_WT_Rep2.cpm.bw
ATAC-seq_heart_E115_mm39_WT_Rep1.cpm.bw
ATAC-seq_heart_E115_mm39_WT_Rep2.cpm.bw
ATAC-seq_heart_E105_mm39_WT_Rep1.cpm.bw
HiC_heart_HH22_galGal6_WT_Rep1_R1_001.fastq.gz
HiC_heart_HH22_galGal6_WT_Rep2_R1_001.fastq.gz
HiC_heart_HH22_galGal6_WT_Rep1_R2_001.fastq.gz
HiC_heart_HH22_galGal6_WT_Rep2_R2_001.fastq.gz
RNA-seq_heart_HH22_galGal6_WT_Rep1_R1_001.fastq.gz
RNA-seq_heart_HH22_galGal6_WT_Rep1_R2_001.fastq.gz
RNA-seq_heart_HH22_galGal6_WT_Rep2_R1_001.fastq.gz
RNA-seq_heart_HH22_galGal6_WT_Rep2_R2_001.fastq.gz
RNA-seq_heart_HH24_galGal6_WT_Rep1_R1_001.fastq.gz
RNA-seq_heart_HH24_galGal6_WT_Rep1_R2_001.fastq.gz
RNA-seq_heart_HH24_galGal6_WT_Rep2_R1_001.fastq.gz
RNA-seq_heart_HH24_galGal6_WT_Rep2_R2_001.fastq.gz

Genome browser session
(e.g. UCSC)

https://genome-euro.ucsc.edu/s/mphan236/heart_chromatin_mm39
https://genome-euro.ucsc.edu/s/mphan236/heart_chromatin_galGal6

Methodology

Replicates

all ChIP, ATAC and RNA-seq experiments were performed in two biological replicates per sample, species, and developmental stage

Sequencing depth	All libraries were sequenced with 100bp pair-end. Sequencing depth for each biological replicate was 100 million fragments for ATAC-seq and 30-50 million fragments for ChIPmentation.
Antibodies	H3K4me1 (Diagenode #C15410037), H3K4me3 (Merck-Millipore #07-473), H3K27ac (Diagenode #C15410174)
Peak calling parameters	ATAC peak calling was done using Genrich v0.6.1 using '-j' mode and default parameters
Data quality	Aligned reads with MAPQ <10 were excluded. Duplicated, unmapped, and unpaired reads were also filtered out.
Software	- samtools v1.10<br>- bowtie2 v.2.3.5<br>- deepTools<br>- BWA-mem v0.7.17<br>- cutadapt v 1.10<br>- Genrich v0.6.1 https://github.com/jsh58/Genrich<br>- Picard v2.23.4
